# Powerful anti-colon cancer effect of modified nanoparticle-mediated IL-15 immunogene therapy through activation of the host immune system: Erratum

**DOI:** 10.7150/thno.39082

**Published:** 2019-08-17

**Authors:** Xiaoxiao Liu, Yanyan Li, Xiaodong Sun, Yagmur Muftuoglu, Bilan Wang, Ting Yu, Yuzhu Hu, Lu Ma, Mingli Xiang, Gang Guo, Chao You, Xiang Gao, Yuquan Wei

**Affiliations:** 1 Department of Neurosurgery and Institute of Neurosurgery, State Key Laboratory of Biotherapy, West China Hospital, West China Medical School, Sichuan University/Collaborative Innovation Center for Biotherapy, Chengdu, 610041, China.; 2 Department of Radiation Oncology, Cancer Center, Affiliated Hospital of Xuzhou Medical University; Jiangsu Center for the Collaboration and Innovation of Cancer Biotherapy, Cancer Institute, Xuzhou Medical University, Xuzhou, 221000, China.; 3 Department of Pharmacy, West China Second University Hospital of Sichuan University, Chengdu, 610041, China.; 4 Department of radiation oncology, Fudan University Shanghai Cancer Center; Department of Oncology, Shanghai Medical College, Fudan University, Shanghai 200032, China; 5 Stanford University School of Medicine, Stanford, CA 94305, USA

In our paper 1 and the supplementary materials, Figure 10C and Supplementary Figure 5 should be corrected as the following Figure A1 and Figure A2.

## Figures and Tables

**Figure A1:**
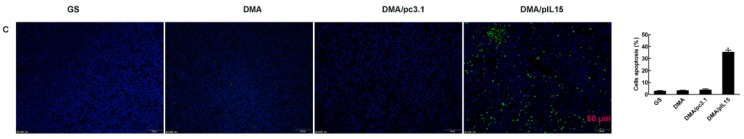
** (Figure** **10C). Detection of cell apoptosis.** Cell apoptosis was assessed by counting the number of TUNEL-positive cells in the field, and a greater level of apoptosis was observed in the DMA/pIL15 group compared to the other groups (mean ± SEM; five high power fields per slide; *, p < 0.01 DMA/pIL15 versus GS, DMA, DMA/pc3.1).

**Figure A2:**
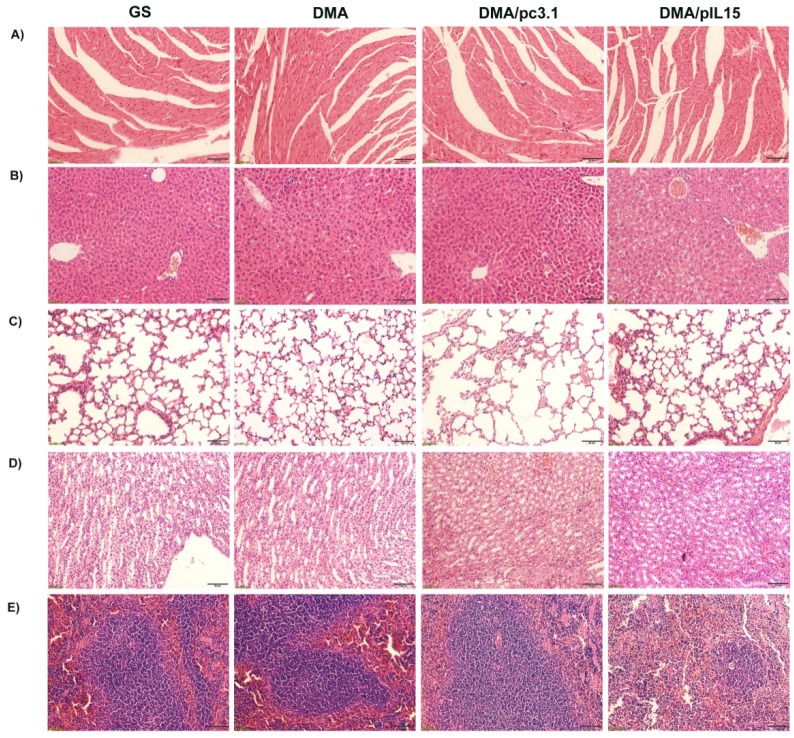
** (Supplementary Figure** **5). Toxicity assessment in vivo with pathological sections.** Histological examinations of H&E-stained sections of vital organs from mice in the different treatment groups: **(A)** heart, **(B)** liver, **(C)** lung, **(D)** kidney, and **(E)** spleen. No significant pathological changes were detected.
